# Pressure redistributing in-seat movement activities by persons with spinal cord injury over multiple epochs

**DOI:** 10.1371/journal.pone.0210978

**Published:** 2019-02-13

**Authors:** Stephen Sprigle, Sharon Eve Sonenblum, Chen Feng

**Affiliations:** 1 Rehabilitation Engineering and Applied Research Lab, Georgia Institute of Technology, Atlanta, Georgia, United States of America; 2 H. Milton Stewart School of Industrial & Systems Engineering, Georgia Institute of Technology, Atlanta, Georgia, United States of America; University of Illinois at Urbana-Champaign, UNITED STATES

## Abstract

Pressure ulcers, by definition, are caused by external forces on the tissues, often in the regions of bony prominences. Wheelchair users are at risk to develop sitting-acquired pressure ulcers, which occur in the regions of the ischial tuberosities, sacrum/coccyx or greater trochanters. As a means to prevent pressure ulcers, instruction on performing pressure reliefs or weight shifts are a part of the rehabilitation process. The objective of this study was to monitor the weight shift activity of full-time wheelchair users with acute spinal cord injury over multiple epochs of time in order to determine consistency or routine within and across epochs. A second objective was to evaluate the accuracy of self-reported pressure relief frequency within each measurement epoch. A wheelchair in-seat activity monitor was used to measure weight shifts and other in-seat movement. The data was classified into multiple in-seat activity metrics using machine learning. Seventeen full-time wheelchair users with spinal cord injury were measured within multiple epochs, each lasting more than 1 week. Across all in-seat activity metrics, no consistent pattern of activity changes emerged. None of the in-seat activity metric changed in any one direction across a majority of subjects. Subjects tended to over-estimate their frequency of performing pressure reliefs. Self-reported pressure relief behaviors are not reliable, and therefore, cannot be used to evaluate preventative behaviors either clinically or within research. This study had the capability of fully investigating in-seat movements of wheelchair users. The results indicated that in-seat movement does not reflect a routine, either in pressure reliefs, weight shifts or other functional in-seat movements. This study has illustrated the complexity of assigning causation of pressure ulcer occurrence to seated behaviors of wheelchair users and identifies the need for improved clinical techniques designed to develop routine behaviors to prevent pressure ulcers.

## Introduction

Pressure ulcers, by definition, are caused by external forces on the tissues, often in the regions of bony prominences. Wheelchair users are at risk to develop sitting-acquired pressure ulcers, which occur in the regions of the ischial tuberosities, sacrum/coccyx or greater trochanters. Pressure ulcers are acknowledged as a significant secondary complication in wheelchair users, and their prevalence of pressure ulcers has been well-documented [1, 2].

Formative research into pressure ulcer etiology using animal models demonstrated that the damaging effects of pressure are related to both its magnitude and duration [3, 4]. Moreover, these controlled studies inducing pressure damage indicated that loading magnitude and duration were inversely related, meaning that tissues can withstand higher loading for relatively short periods of time compared to lower tissue loads. Based upon this and related work, clinical interventions have been based upon the premise that both the magnitude and duration of loading are important. Wheelchair users are taught pressure relief maneuvers and weight-shifting activities as a means to reduce load duration on tissues. Instruction on performing pressure reliefs or weight shifts are a routine part of the rehabilitation process. Clinical guidelines vary somewhat, but collectively, recommend that persons perform pressure reliefs for 15 to 60 seconds every 15 to 60 minutes [5–8].

Over the years, a variety of techniques have been used to assess adherence to these guidelines. A few studies utilized objective measurements of weight shift activity but utilized disparate operational definitions. Most utilized complete offloading of the sitting surface [9–11], whereas more recent studies reported weight-shift activity using a range of parameters that included reduced loading or changes in center of pressure on the seat surface [12, 13]. Regardless of the definition, studies using objective reporting of weight-shift activity indicate that persons do not adhere to the rehabilitation regimen as taught.

The objective of rehabilitation training is to formulate pressure reliefs as routine or automatic. Developing routines as a part of improving healthy behaviors has received much attention recently as means to manage chronic illness or avoid complications [14–17]. One characteristic of routine behavior is documenting the regularity [15] of a particular action or behavior. In the context of pressure ulcer prevention, little is known about the automaticity or routine of pressure reliefs and weight shift activities, especially over time. Documenting a routine or lack thereof, is the first step in devising training strategies that target the prevention of pressure ulcers and in designing studies capable of associating behaviors with pressure ulcer occurrence.

The objective of this study was to monitor the weight shift activity of full-time wheelchair users with acute spinal cord injury over multiple epochs of time in order to determine consistency or routine within and across epochs. A second objective was to evaluate the accuracy of self-reported pressure relief frequency within each measurement epoch.

Within this paper, the term weight shifts will refer to in-seat activities, whether volitional or not, that result in a change in force on the buttocks tissue that may have a beneficial effect on tissue health. A controlled study using three different cushions demonstrated that ischial blood flow increases were achieved during weight shift maneuvers consisting of forward and side leans [18]. The term pressure relief will be limited to volitional maneuvers, such as those taught during rehabilitation. As such, all pressure reliefs are included in weight shift activity, but not all weight shifts can be considered pressure reliefs.

## Methods

Full-time wheelchair users with spinal cord injury were recruited for the study. Subjects were recruited within their first year after completing rehabilitation following injury. Inclusion criteria included being an adult and using a wheelchair as his or her primary means of mobility. Participants were excluded if they reported ambulating within the home or if they had a condition that limited how long they could sit in their wheelchairs. All subjects provided informed consent to participate in the research protocol as approved by the Institutional Review Board of the Georgia Institute of Technology. Additional approval was received from the U.S. Army Medical Research and Materiel Command (USAMRMC), Office of Research Protections (ORP), Human Research Protection Office (HRPO).

**Fig 1:**
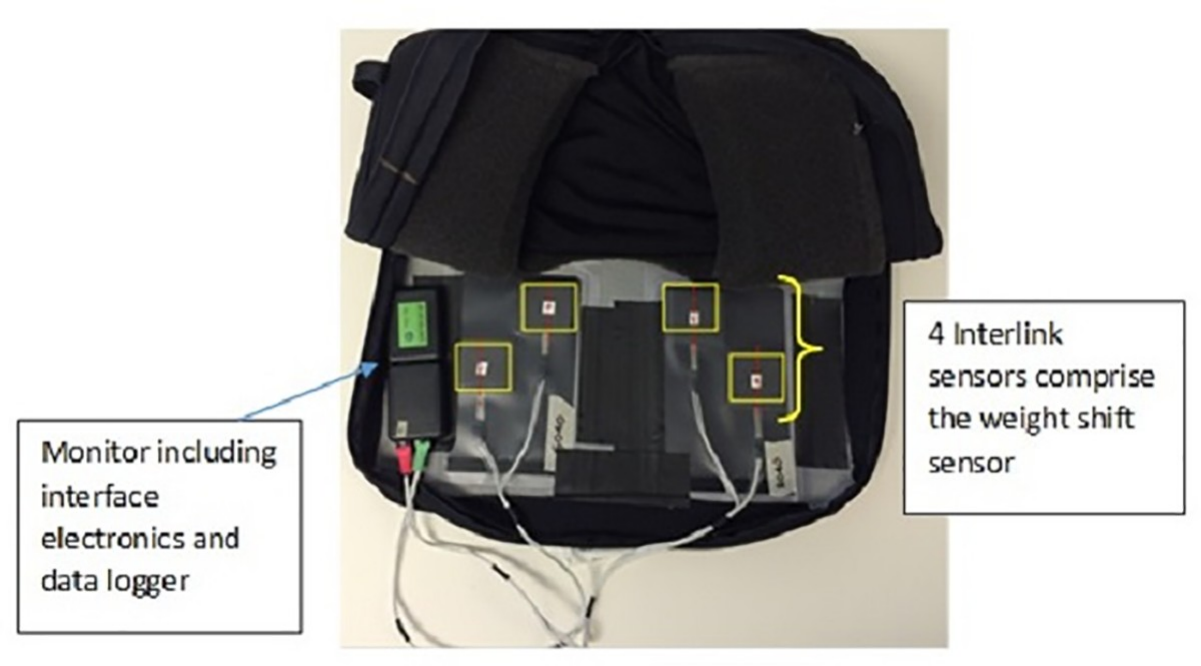
Weight shift monitoring system in position within cushion cover.

The Wheelchair In-seat Activity Monitor (WiSAT) consists of a data logger and seat sensor (Fig 1). The seat sensor is comprised of four 50 mm square Interlink FSR force sensors arranged in a trapezoidal pattern and positioned under the cushion. The data logger collected voltages from each sensor at 1 Hz. Each sensor was calibrated using bench tests to determine the voltage-force curves. Both WiSAT components were housed within the wheelchair cushion cover.

During instrumentation of the wheelchair, an initialization protocol was deployed to establish the relationships between interface pressure- measured at the buttocks-cushion interface- and the four seat sensors measured underneath the cushion. A BodiTrak pressure measurement system (Vista Medical) was used for the protocol and was calibrated according to the manufacturer’s instructions. Subjects were led through a series of leans, reaches and in-seat movements while pressure and force data was collected above and below the cushion, respectively. This initialization protocol collected a training data set for each subject. This individual assessment was needed because pressures on the cushion and changes in force due to movement will differ across people and cushions.

This training data set was used to develop an individualized classifier. The classifier interprets the measured forces to output the 3 core in-seat metrics: pressure reliefs, weight shifts and center of pressure (CoP) displacement. The magnitude of unloading, used to classify pressure reliefs and weight shifts using a knn classifier, was determined by associating seat sensor features with ground truth status using interface pressures from the training set measurements in an approach previously reported [19]. Similarly, CoP displacement was calibrated for each subject by determining the relationship between the CoP displacements as measured on the interface pressure mat and on the seat sensor during training. The seat sensor CoP displacement was then scaled according to this relationship.

After initialization, the WiSAT was deployed to collect data for about 2 weeks while subjects went about their daily activities. For this study, subjects were monitored over multiple epochs separated by at least a few months. Subjects did not have to engage the WiSAT during the measurement epoch.

During the visits to set-up the instrumentation, participants were asked several questions about their equipment, equipment use and aspects of their lives. Included in these questions was a request for the participants to estimate the frequency of performing pressure reliefs as ‘every 15 min’, ‘every 15–30 min’, ‘between 30–60 min’ or less than once per hour. These estimates were compared to actual measurements of in-seat activity. Two comparisons were made, one to the number of pressure reliefs performed and the second to the number of weight shifts.

### In-seat activity metrics

Three core variables were defined and identified using the classification algorithm:

*Pressure relief (PR)* is defined as a reduction of seat loading by 90% for between 15–120 secs. (Durations exceeding 120 secs were considered transfers out of the chair.)

*Weight shift (WS)* is defined as a movement that would unload either or both ischia by at least 30 percent for at least 15 secs [12, 20].

*COP displacement*: The displacement of CoP was calculated as the square root of the sum of the squares of the finite differences of the absolute medial-lateral and anterior-posterior CoP locations summed over a 5-sec window.

WSs and PRs exhibited episodic activities which could be counted and assigned a frequency of occurrence. As indicated, the difference between the two is embodied by both magnitude and duration of pressure reduction. Based upon their respective definitions, all PRs were also classified as WSs.

CoP displacement is a measure of in-seat movements embodied by changes in posture such as leaning, reaching and other postural shifts. CoP displacement includes both transient movements as well as shifts in posture that may be maintained for longer durations.

These three core variables were then used to calculate the in-seat activity metrics for analysis:

TimeInChair: *the amount of time that the chair is occupied each day in hours*.

PRFrequency: *the mean number of PRs per occupied hour each day*.

WSFrequency: *the mean number of weight shifts per occupied hour each day*.

MaxTimeBetweenEvents: *maximum time in minutes between an unweighting event such as a WS or out-of–chair transfer*

FreqActiveSegments: *Frequency of segments of time (per occupied hour each day) with a drop in load ≥ 30% and/or sufficient CoP movement to be defined as ‘active’*. *To be defined as active*, *the CoP of the body on the wheelchair cushion must meet the threshold of moving >10 cm over a 5 second window*.

%TimeReducedLoad: *Percent of occupied hours of the day with total load reduced by at least 30% (weight shift threshold)*

To be included in the analysis, a day of monitoring was required to have a minimum wheelchair occupancy of 4 hours. Daily measurements were then averaged across all valid days within the measurement epoch.

The mean and standard deviation of in-seat activity variables were calculated for each epoch and were subsequently used to calculate the coefficient of variation (CoV), representing the relative dispersion of values across subjects. For each individual subject, the mean and standard deviation from each measurement epoch were calculated. These values were subsequently used to calculate standard error of the measurement (SEM).

Differences over time were characterized by comparing the mean values of subsequent epochs. A parameter was considered different across epochs if the difference between the mean values exceeded the SEM of the preceding epoch. “No change” was defined if the mean fell within the mean ±SEM of the preceding epoch. For the 8 subjects measured twice, the difference in each parameter was labeled as ‘increasing’, ‘decreasing’ or ‘no change’. For the 9 subjects monitored more than twice, parameters were labeled as ‘increasing’, ‘decreasing’, ‘no change’, or ‘reciprocal’. Reciprocal was defined as a parameter that demonstrated both increases and decreases over the measurement epochs.

## Results

### Subjects

Seventeen subjects were enrolled in the study. All subjects had a spinal cord injury and were enrolled in the study within their first year post-discharge from rehabilitation. All were monitored more than once with nine subjects monitored more than twice.

Participants were predominantly middle-aged, white, male wheelchair users (Table 1). The average age at their first visits was 32 years old (std dev 11 years), 12 (71%) of them were white, and the number of male participants was 13 (76%). Participants exhibited a range of injury levels and functional presentations. Most were not able to walk two steps (n = 15 (88%)), and 9 (53%) had some sensation in their buttocks region. Only a few of them had attended or completed college (n = 6 (35%)).

**Table 1:** Participant demographic information.

Characteristic	Participants (N = 17)
**Age-years, mean (Std dev)**	32 (13)
**Male, no. (%)**	13 (76.5)
**Race, no. (%)**	
Other	4 (23.5)
Black/African American	1 (5.9)
White	12 (70.6)
**Level of Injury, no. (%)**	
Cervical (C5-C8)	4 (23.5)
Upper Thoracic (T1-T6)	7 (41.2)
Lower Thoracic/Lumbar (T7-L1)	6 (35.3)
**Occupation, no. (%)**	
Paid/Unpaid employment	11 (64.7)
Unemployed	4 (23.5)
Student	1 (5.9)
NA	1 (5.9)
**Education, no. (%)**	
Attended or completed HS	11 (64.7)
Attended or completed college	6 (35.3)
**Complete injury?, no. (%)**	
Yes	11 (64.7)
No	6 (35.3)
**Ambulation, no. (%)**	
Non-ambulatory	15 (88.2)
Able to ambulate (at least 2 steps)	2 (11.8)
**Sensation, no. (%)**	
Yes	9 (52.9)
No	8 (47.1)
**Spasticity, no. (%)**	
Regularly throughout the day	2 (11.8)
A few times per day	8 (47.1)
Not every day, several times during the week	1 (5.9)
Rarely, if ever	6 (35.3)
**Living Alone, no. (%)**	
Yes	1 (5.9)
No	16 (94.1)

Subjects were scheduled to be monitored for about 2 weeks with the average epoch lasting 12.5 days (std dev = 4.4). On average, the first monitoring epoch or period occurred 110 days (range: 1–340 days) after discharge from the rehabilitation hospital. The timespan between subsequent monitoring epochs averaged 152 days (range: 94–287 days). While the intent was to monitor subjects multiple times within a year, scheduling issues resulted in disparate timeframes between epochs.

### Summary of in-seat activity parameters within each epoch

Table 2 includes the descriptive parameter values across the subjects within each measurement epoch for in-seat activity. Of the six in-seat movement metrics, five are configured so an increase reflects a more desirable level of activity. Only MaxTimeBetweenEvents is configured differently, with an increase in duration being less desirable.

**Table 2:** In-seat activity parameters across measurement epochs.

Variable	Epoch	Subject N	Mean	StDev	CoV (%)
TimeInChair (hr)	1	17	8.0	1.7	21
	2	17	8.4	2.2	26
	3	9	9.0	2.5	28
	4	4	10.0	3.6	36
PRFrequency (per hour)	1	17	0.52	0.43	82
	2	17	0.51	0.38	74
	3	9	0.36	0.26	72
	4	4	0.22	0.26	115
WSFrequency (per hour)	1	17	3.3	3.9	117
	2	17	2.8	2.0	73
	3	9	2.4	1.9	80
	4	4	1.5	0.6	39
MaxTimeBetweenEvents (min)	1	17	106.2	47.7	45
	2	17	132.4	81.2	61
	3	9	146.5	84.7	58
	4	4	190.9	69.9	37
FreqActiveSegments (per hour)	1	17	62.2	39.2	63
	2	17	74.5	39.7	53
	3	9	76.8	33.1	43
	4	4	72.6	44.1	61
%TimeReducedLoad (%)	1	17	3.9	2.2	55
	2	17	4.0	2.4	60
	3	9	3.1	1.5	49
	4	4	2.6	1.7	66

Data indicate that subjects spent substantial amounts of time in their wheelchairs, exceeding 8 hours per day. In-seat activity metrics exhibited a wide range across subjects, as indicated by the coefficient of variation (CoV).

Subjects performed PRs at a rate of about once every 2 hours or less with weight shifts performed about 5–6 times more often than PRs at each epoch, on average. Episodes of active seating were much more prevalent, occurring about 60–75 times per hour. This indicates that while activity was prevalent, the active sitting episodes were of short duration. The percentage of time that persons sat with decreased load was only about 3–4% of the time.

### Differences in in-seat activity

Table 3 lists the differences in in-seat activity over the measurement epochs. No consistent pattern emerged as evidenced by the absence of any in-seat activity metric changing in any direction across a majority of subjects. The majority of subjects monitored 3 or more times demonstrated a reciprocal pattern of activity, meaning that the changes in metrics rose and fell over subsequent epochs.

**Table 3:** Differences in in-seat activity over time across subjects.

***Subjects with 2 measurement epochs*** (n = 8)				
***Variables***	Number Increasing	Number Decreasing	Number Unchanged	
TimeInChair	2	4	2	
PRFrequency	3	4	1	
WSFrequency	5	3	0	
MaxTimeBetweenEvents	4	2	2	
FreqActiveSegments	4	2	2	
%TimeReducedLoad	3	5	0	
***Subjects with 3+ measurement epochs*** (n = 9)				
***Variables***	Number Increasing	Number Decreasing	Number Unchanged	Number Reciprocating
TimeInChair	5	1	0	3
PRFrequency	3	4	0	2
WSFrequency	1	1	0	7
MaxTimeBetweenEvents	1	0	0	8
FreqActiveSegments	0	2	0	7
%TimeReducedLoad	1	3	0	5

**Fig 2:**
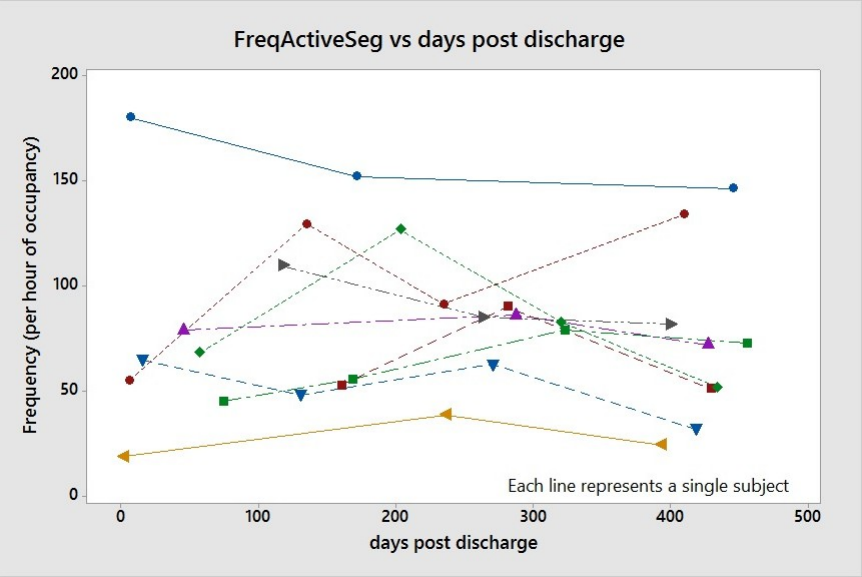
Scatter plot of frequency of active segments over days post discharge.

As an example of the results, Fig 2 depicts frequency of active segments versus the days post discharge from rehabilitation for the subjects monitored 3 or more times. This figure reflects the tabulated data and illustrates that 7 of the 9 subjects reflected reciprocal changes over time which was the dominating outcome for this in-seat activity metric.

### Accuracy of estimating pressure reliefs

**Fig 3:**
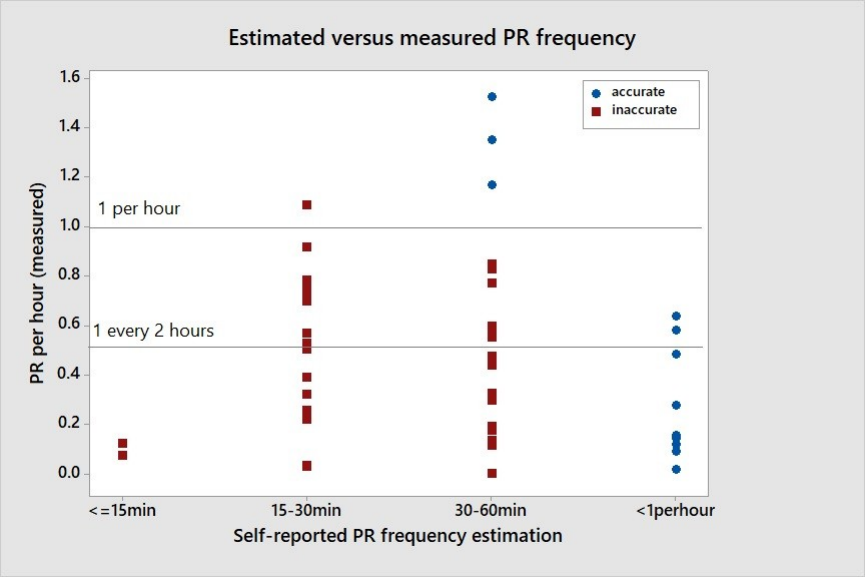
Estimated versus measured PR frequency at each epoch.

Subjects were asked to estimate frequency of pressure reliefs during each visit, so the 17 subjects reported estimations a total of 47 times. Of the 17 subjects, 11 offered estimates of PR frequency that changed across subsequent epochs. Ten subjects estimated correctly during at least one epoch. However, 8 of those 10 estimated less than 1 PR per hour. Two subjects estimated and performed a PR more than once per hour. Across all 47 epochs of measurement, 34 (72%) reflected an overestimation of PR frequency. Fig 3 illustrates these estimations which indicates that most of the accurate estimations were for PR frequencies of less than once per hour.

**Fig 4:**
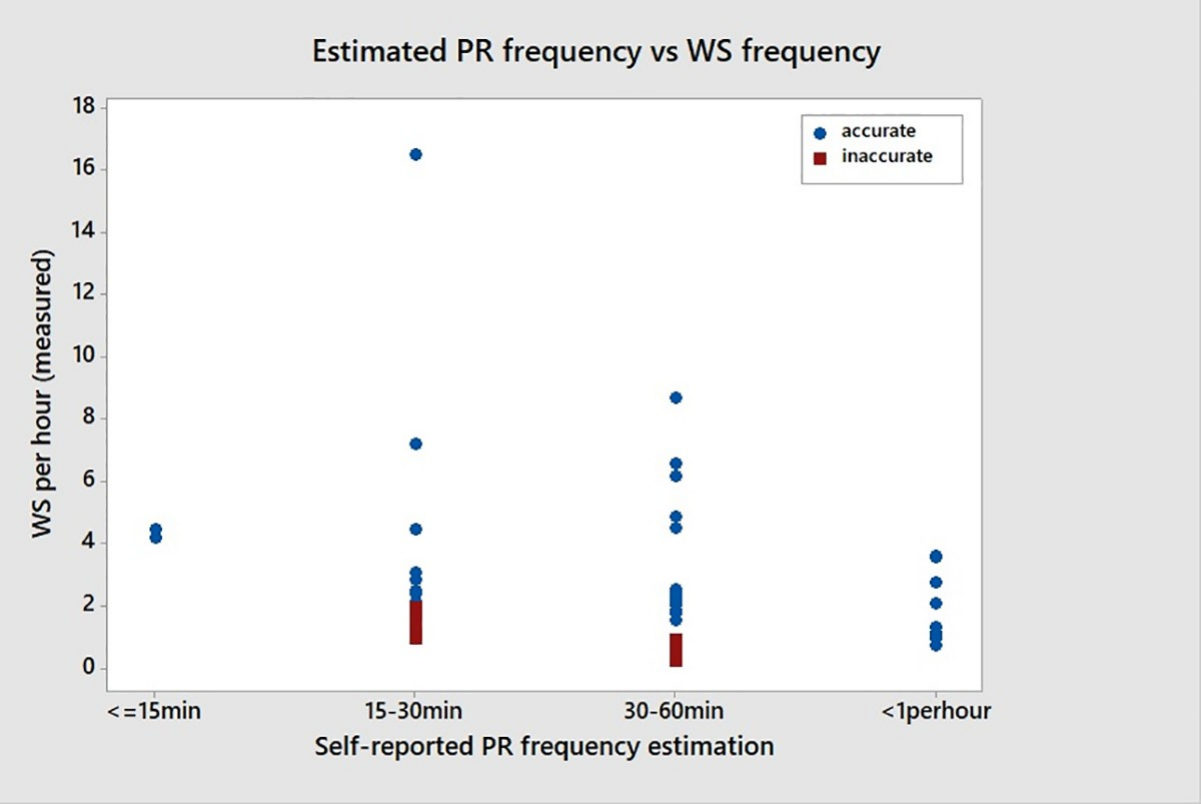
Estimated PR versus measured WS at each epoch.

The self-reported PR frequency was also compared to WS frequency, a metric which includes PRs, by definition. This comparison allowed assessment of how well people characterized overall postural shifts embodied by volitional pressure reliefs as well as other maneuvers classified as weight shifts. Overall, the estimations of individuals were more accurate, 34 of the 47 epochs (72%). Sixteen subjects estimated performing a PR more frequently than once per hour during at least one epoch and 14 exceeded that estimate when considering WSs. Fig 4 illustrates the estimated pressure reliefs relative to the measured WS during each epoch.

## Discussion

With respect to pressure ulcer prevention, in-seat activity metrics relate to the duration of loading because they reflect measures of pressure redistributing events or activities. Within the metrics, only the frequency of PRs is tied to a specific rehabilitation activity. Regular PRs are taught as a behavior intended to prevent pressure ulcer occurrence. However, fuller assessment about in-seat activity that encompasses both PRs as well as other postural shifts that affect loading can add to our knowledge about preventative activities and behaviors. This study focused on both objective and self-reported measures over different measurement epochs so is able to draw inferences about routine behaviors over time.

When considering this data in relation to the in-seat variables and self-reporting, one can draw some inferences.

1. The daily in-seat behavior of people varies widely, both within a person and across persons
2. In-seat activity behavior does not reflect a consistent trend over time, either within a person or across people
3. Participants offer poor estimates of performing pressure reliefs but their estimates are more closely aligned with frequency of performing weight shifts.

Stockton et al. asked wheelchair users of various diagnoses to estimate the frequency of performing pressure reliefs and found that 24% reported performing a pressure relief more than once per hour and 13% reported not performing them at all. The remaining subjects estimated performing reliefs every 1 to 5 hours [21]. Another study using self-report found that about 50% of wheelchair users with SCI stated that they “often” or “always” performed a pressure relief every 30 minutes [22]

The current study collected self-report estimations at each epoch. All of the subjects went through rehabilitation at two facilities with both educating their patients to perform pressure reliefs at least every 30 minutes. Overall, the participants who performed very few PRs were accurate at self-report estimations of PR behavior. The majority of subjects provided different estimations of PR frequency across the different epochs. This result indicates that the subjects did not view their PR frequencies as routine over subsequent epochs. Fourteen subjects estimated performing pressure reliefs less than once every 30 minutes during at least one epoch. This finding offers evidence that a large percentage of the subjects admit to performing PRs at a lower frequency than taught during rehabilitation.

Overall, the results of this study are consistent with prior studies utilizing self-reported PR frequency. Specifically, a majority of persons overestimate their frequency of performing pressure reliefs. Because this study also measured actual PR frequency, a more robust assessment is possible. Most informative was that estimations of PR frequency were more accurate when considering WS activities. While not all weight shifts are volitional, persons might include such postural shifts during their self-assessment of in-seat movements.

The data clearly indicated that subjects did not adhere to the taught rehabilitation regimen. Subjects had been taught to perform pressure reliefs every 30 minutes. Overall, subjects averaged about 1 PR every two hours. This low frequency of volitional pressure reliefs is consistent with other studies of community-living wheelchair users [9, 12]. The other in-seat activity variables are dominated by everyday sitting behavior. Weight shifts and active sitting metrics reflect postural changes due to many activities such as reaching, leaning or squirming. Sitting is a functional posture for wheelchair users so these other in-seat activity variables will reflect functional activities that impart changes to the loading on the seat surface.

The data suggest that episodes of active sitting (FreqActiveSegments) were common, averaging 60 episodes per hour. This metric includes changes in COP that result from postural shifts as well as reduced loading. However, active sitting translated to a relatively low percentage of time in sitting with reduced buttocks loading. On average, subjects sat with reduced load 4% or less of the time. Moreover, the maximum duration of time that a subject sat between an unweighting event (WS or out-of-chair transfer) was quite lengthy, exceeding 100 minutes.

The relatively wide daily variation of these metrics within subjects and the lack of consistent behavior measured at different times indicate that in-seat movement do not reflect a routine or development of routine over time. The subjects in this study were monitored in the year following discharge from rehabilitation. Because each was trained in a pressure relief regimen, monitoring over multiple epochs permitted assessment of routine or automaticity in weight shifting behaviors. Overall, subjects did not exhibit a trend in changes of in-seat activity over time. Some persons increased their desirable in-seat activity while others decreased. Of the 17 subjects, 9 were monitored during 3 or 4 epochs. This cohort permitted better assessment of trending over time, but did not reflect any consistent changes. In fact, the data showed that the majority of these subjects demonstrated a reciprocal pattern of activity changes. This result suggests that weight-shifting routines are not established after discharge from rehabilitation.

Furthermore, defining a routine behavior can be inferred as difficult within a changing context. After returning to home, persons with spinal cord injury are faced with myriad issues that might impact developing a routine with respect to pressure ulcer prevention. For instance, changes in equipment or home environment, health, or job status are a few situations that can impact functional activities and, by extension, in-seat behaviors.

The in-seat metrics, except for PR frequency, are based upon functional movements. The lack of consistent in-seat activity reflects a hypothesis that functional activities change from day to day. The lack of routine in performing pressure reliefs is a potentially important issue that deserves thought and reflection. If functional movements contribute to unweighting buttocks tissue, they should be facilitated. This underscores the importance of positioning people in functional postures from which they can perform functional activities. The results also complicate the study of in-seat behavior in relation to pressure ulcer formation. Because functional movement is comingled with volitional, and infrequent, pressure reliefs, extracting the influence of only pressure reliefs on tissue health becomes complicated. By measuring other metrics that reflect changes in loading, a more complete analysis of the forces at the buttock-cushion interface is possible.

The in-seat activities analyzed in this study impacted tissue loading. The finding that they were all highly variable can be used to inform interventions and research that target pressure ulcer prevention. First, the results suggest that pressure redistributing activities need to include more than volitional pressure reliefs. Other pressure redistributing events including weight shifts and active sitting are much more prevalent. A controlled study of in-seat movements found both an increase in blood flow and a decrease in interface pressure during relatively small postural changes resulting from leaning and reaching [18]. The frequency of weight shifts documented in this study in combination with results from the controlled study suggest that weights shits and active sitting should not be discounted as potentially contributing to prevention.

Clinically, the results indicate that routine weight-shifting behaviors are not being developed during rehabilitation training. Different strategies may be needed to develop weight-shift routines during and after rehabilitation. Researchers [14, 15, 17] describe routine as a more complex construct than simply replicating a behavior. Developing routine requires repetition and practice, but can be affected by external stimuli and associations with other factors (i.e. time, location, goals) [17].

From a research perspective, the results illustrate the challenge (and limitation) in cross-sectional methods that utilize a single measurement epoch. The wide variation of metrics within persons, can be inferred to mean that in-seat activity and behavior might not be stable enough to determine a person’s typical in-seat activity within a single and relatively short epoch.

Research underway in behavioral change techniques [16] may have application in the prevention of pressure ulcers within clinical interventions and research methodologies. Adopting behavioral change techniques represents a design opportunity to develop technology that can serve as a means to encourage routine preventative behaviors. Such technology may function in a manner similar to activity monitors that are becoming more ubiquitous in the mainstream consumer market. This technology would permit long term monitoring with features designed to change behaviors. In addition to its clinical purpose, such measurement will generate enough data to begin to better understand the influence of postural changes and pressure redistribution in relation to PU occurrence in a large number of people.

### Limitations

The data set was collected on a small cohort of full-time wheelchair users with spinal cord injury, so one cannot definitively conclude that it represents the general population of wheelchair users who are at risk of pressure ulcers. The imbalance of the times between measurement epochs does not allow for a clean assessment of trends over time. The measurements taken over two or more epochs, however, does not allow one to infer that any particular epoch was reflective of steady-state behavior. Therefore, drawing conclusions about changes in behavior over time is not possible with this dataset.

### Conclusion

This study had the capability of fully investigating in-seat movements of wheelchair users that included unweighting the sitting surface as well as re-distributing loads over the sitting surface. Multiple measurement epochs indicated that in-seat movement does not reflect a routine, either in pressure reliefs, weight shifts or other functional in-seat movements. Relatedly, self-reported PR behaviors are not reliable, and therefore, cannot be used to evaluate preventative behaviors either clinically or within research. This study has illustrated the complexity of the problem in assigning causation of pressure ulcer occurrence to seated behaviors of wheelchair users and identifies the need for improved clinical techniques designed to develop routine behaviors to prevent pressure ulcers.
